# Return-to-performance in elite soccer players after Achilles tendon ruptures: a study using a weighted plus/minus metric and matched-control analysis

**DOI:** 10.1007/s00167-023-07607-5

**Published:** 2023-10-18

**Authors:** Pedro Diniz, Diogo Lacerda, Bruno Mendes, Hélder Pereira, Frederico Castelo Ferreira, Gino M. M. J. Kerkhoffs

**Affiliations:** 1 Department of Orthopaedic Surgery, Hospital de Sant’Ana, Rua de Benguela, 501, 2775-028 Parede, Portugal; 2 Department of Bioengineering and iBB-Institute for Bioengineering and Biosciences, Instituto Superior Técnico, Universidade de Lisboa, Lisbon, Portugal; 3 Associate Laboratory i4HB-Institute for Health and Bioeconomy, Instituto Superior Técnico, Universidade de Lisboa, Lisbon, Portugal; 4 Fisiogaspar, Lisbon, Portugal; 5 Fulham Football Club, London, UK; 6 Orthopaedic Department, Centro Hospitalar Póvoa de Varzim, Vila do Conde, Portugal; 7 Ripoll y De Prado Sports Clinic: FIFA Medical Centre of Excellence, Murcia-Madrid, Spain; 8 University of Minho ICVS/3B’s-PT Government Associate Laboratory, Braga/Guimarães, Portugal; 9 Department of Orthopaedic Surgery, Amsterdam Movement Sciences, Amsterdam University Medical Centers, Amsterdam, The Netherlands; 10 Academic Center for Evidence Based Sports Medicine (ACES), Amsterdam, The Netherlands; 11 Amsterdam Collaboration for Health and Safety in Sports (ACHSS), Amsterdam, The Netherlands

**Keywords:** Achilles tendon, Tendon rupture, General sports trauma, Football (soccer), Epidemiology, Statistics

## Abstract

**Purpose:**

Studies have shown decreased match participation and shortened careers in athletes suffering Achilles tendon ruptures (ATRs), but assessment using a true performance metric is lacking. Plus/minus (PM) metrics provide a practical and objective approach to player performance assessment and are commonly used in other sports. This study aimed to quantify and compare individual player performance variations in elite football league players who sustained ATRs and returned to play within 1 year compared to those without ATRs, using a PM metric.

**Methods:**

Player and team data were sourced from Transfermarkt.com. Male players sustaining ATRs between 2007 and 2018 were identified through injury reports. A control group (CTRL) was matched by position, age, height, and league, with a 6:1 ratio of controls to ATR subjects. The day of injury was considered “time zero”. Year -1 corresponds to the 360 days preceding injury, and Year 1 to the interval between 360 and 720 days after. Performance in the player’s main team was evaluated using a previously validated weighted PM metric. Only data from Year -1 and Year 1 were used for ATR versus CTRL group comparisons. Statistical significance was set at *p* < 0.05.

**Results:**

The ATR group included 125 athletes. Data from more than 76,000 matches were analyzed. No statistically significant differences in net weighted PM metric between Year -1 and Year 1 were found.

**Conclusion:**

No differences were found between athletes suffering from ATRs and controls regarding the weighted PM metric.

**Level of evidence:**

III.

## Introduction

Achilles tendon ruptures (ATRs) can have a devastating impact on an elite soccer player’s career. Previous research has shown that a significant portion of athletes suffering from these injuries shows poor sports performance after return-to-play (RTP) [2, 8, 29, 31], manifesting as decreased match participation and shortened careers, which may result from persistent symptoms [6], decreased strength [33], changes in lower limb biomechanics [3], psychological factors [17, 28], and loss of space in the team owning to prolonged time off the pitch.

Because a high caseload of injuries in elite athletes is uncommon in the clinical setting, the use of public sources of information is a common practice in research aimed at assessing the consequences of injuries in this population [2, 4, 5, 8, 19, 20, 24, 29, 31]. Transfermarkt.com is a particularly noteworthy source, and while it is primarily aimed at reporting club transfers, it also contains pertinent data for clinical and sports analytics, such as injury reports, match outcomes, and player match participation data [30]. Furthermore, prior studies have confirmed the accuracy of this website in correctly identifying the type and location of injuries in 89% of instances [21].

Previous studies on ATRs using such an approach have focused mostly on whether players returned to similar competitive levels [2, 8], minutes played per match (MPM) [2, 4, 29, 31], or generic performance markers, such as games started [2, 31], or goals scored [4, 29, 31]. What is currently missing from the literature is using a true performance metric while assessing the consequences of ATRs in elite soccer players. Unfortunately, despite several attempts [15, 22], soccer has no universally accepted individual performance metric.

Plus/minus (PM) metrics provide a practical and objective approach to player performance assessment and are commonly used in other sports, such as American football [24] and basketball [20]. These metrics summarize a player’s positive and negative effects on the match outcome depending on whether they are playing or not [15]. In its simplest form, PM metrics can be calculated by dividing each differential in the target metric occurring in a match, e.g., goals scored, by the corresponding number of minutes that player was on the pitch. In addition, to track the player’s net performance throughout the season, the results of multiple matches may be combined.

Thus, this study aimed to quantify and compare individual player performance variations in elite soccer players who sustained ATRs and returned to play within 1 year compared to those without ATRs, using a PM metric. The null hypothesis was that there would be no differences in player performance between groups, as measured using a PM metric.

## Materials and methods

This study employs a previously reported framework for data acquisition and analysis, with the details reported elsewhere [2]. From January to March 2021, soccer player data with ATRs were sourced from Transfermarkt.com. The selection criteria focused on first or second-league players since the 2007/2008 season with Achilles-related injuries leading to over 90 day absence. Due to COVID-19 disruptions on competition calendars, only injuries before March 31, 2018, were considered, as a 24-month follow-up was required. Players with partial Achilles tears, or those injured outside the primary leagues or without team affiliation, were excluded. Two researchers independently reviewed each entry. Inclusion was limited to players with club reports, press releases, or interviews that specifically mentioned a complete ATR.

Information extracted included date of birth, position, club transfers, match results, number of goals scored and suffered, and their timing in the match. Missing match minute data were addressed using spline interpolation and backfilling resolved missing categorical data on match participation.

The ATR group in the present study consisted of male soccer players who suffered ATRs without major time loss injuries in the first year following rupture. Following previous guidelines for epidemiological studies [9], a major time loss injury was any injury causing a reported absence above 28 days. In addition to the abovementioned exclusion criteria, athletes who did not RTP within 1 year following injury were excluded, as were those who retired or were left without a club in the two years post-ATR.

A matched-control group (CTRL) comprised players of similar playing position, age, and height, and, whenever possible, competing in the same league as the study subject. Six controls per study subject were selected, with the same control allowed in more than one comparison. Non-contemporary controls were also allowed, i.e., players whose data came from seasons different than the experimental subject. Potential control athletes were retrieved from the initial database to screen for ATRs and correspond to active players between 2007 and 2018. Similarly to the ATR group, these athletes were excluded if they suffered a major time loss injury in the timeframe of interest.

As the research was based solely on publicly available data without direct interactions or interventions with individual subjects, specific informed consent was not sought. No personal identifiers or sensitive data were processed beyond what is publicly accessible, and all individual data analyses were anonymous.

### Analysis of match participation

Match participation in the ATR group was compared to the CTRL group. The day of injury was considered “time zero” for both groups; thus, for each subject-control comparison, the same time of year was considered, even if pertaining to different years, ensuring a similar competitive context, namely match congestion and weather conditions. Year -1 corresponds to the 360 days preceding injury, Year 0 to the 360-day interval following injury, and Year 1 to the interval between 360 and 720 days after.

Comparisons were made regarding matches played, matches in which the athlete was in the starting 11, and average and cumulative MPM in 30- and 360-day intervals. Reasons for match absence were categorized into: “medical issues”, “coaching decisions”, and “other”. This categorization was based on the information available from the Transfermarkt.com website and included in the player match participation page. If an injury had been recorded in any match in which the player was not in the squad, it was assumed that the injury caused the player’s absence from the team. If the player sat on the bench but did not play any minutes or if the player was not in the squad and no comment was explicit, it was assumed to be a “coaching decision”. Conversely, if a commentary related, for example, to disciplinary action or a player being called for national team matches, it was categorized as “other”. Comparisons of matches lost due to “medical issues” were performed in absolute and adjusted to match exposure terms, as recommended elsewhere [9].

### Analysis of player performance

We used a modified version of the weighted PM metric proposed by Schultze and Wellbrock [27] to evaluate individual player performance. This metric is calculated on a per-minute basis using the following formula:

1 $${pm}_{x}\left(t,w\right)=\sum\limits_{w=1}^{W}\sum\limits_{t=1}^{T}\left\{\left[\left(\frac{{wp}_{w}^{\mathrm{Opp}}- {wp}_{w}^{\mathrm{Own}}}{{T}_{w}}\right)+\left(\Delta {\mathrm{goals}}_{t,w}- \Delta {\mathrm{goals}}_{t-1,w}\right) \times \left(\frac{2}{1+ \left|\Delta {\mathrm{goals}}_{t,w}\right|- \left|\Delta {\mathrm{goals}}_{t-1,w}\right|}\right)\right] \times {on}_{w,t}^{x}\right\},$$

where *T* is the total number of minutes available in the match, *w* is the week of the game (as in a season has *W* weeks), *t* is the minute in match play, *wp*^Opp^ and *wp*^Own^ are the opposing and the player’s team winning probabilities, respectively, and Δgoals is the goal differential between the opposing and the player’s teams. In the original description of this metric, the researchers used betting quotas from Bet365.com for the team’s winning probabilities. Because the population included in this study represents a worldwide distribution and not all teams and matches could be found in the bookmaker’s offers in a practical way, it was decided to compile all the matches available and perform an empirical calculation of the winning probabilities for each team, depending on whether said team was playing home or away, using a database of more than 500,000 matches. Finally, the whole performance analysis computation is multiplied by 1 if the player is on the pitch or 0 otherwise.

Individual player performance was calculated for all matches played for the athlete’s main team (meaning that matches played for national teams or reserves were excluded from the performance analysis) and summed in 30- and 360-day intervals. Only data from Year -1 and Year 1 were used for ATR versus CTRL group comparisons.

### Statistical analysis

Statistical evaluation was conducted using the Statsmodels and SciPy libraries in Python. Mean and standard deviation values were reported for variables that followed a normal distribution, while median and interquartile range (IQR) were used if otherwise. Depending on the number of groups and data distribution, comparisons were made using Student’s *t* test, Mann–Whitney *U*, or one-way ANOVA. The Shapiro–Wilk test was used to check the normality assumption. The threshold for statistical significance was established at *p* < 0.05. This study did not include a sample size calculation due to the lack of existing data for this specific metric and the non-normal distribution of the data. To address concerns about the robustness of the findings and potential Type II errors, we employed a bootstrap method using 10,000 repetitions to compute the Mann–Whitney *U* test statistic and the Hodges–Lehmann shift estimator.

## Results

**Fig. 1:**
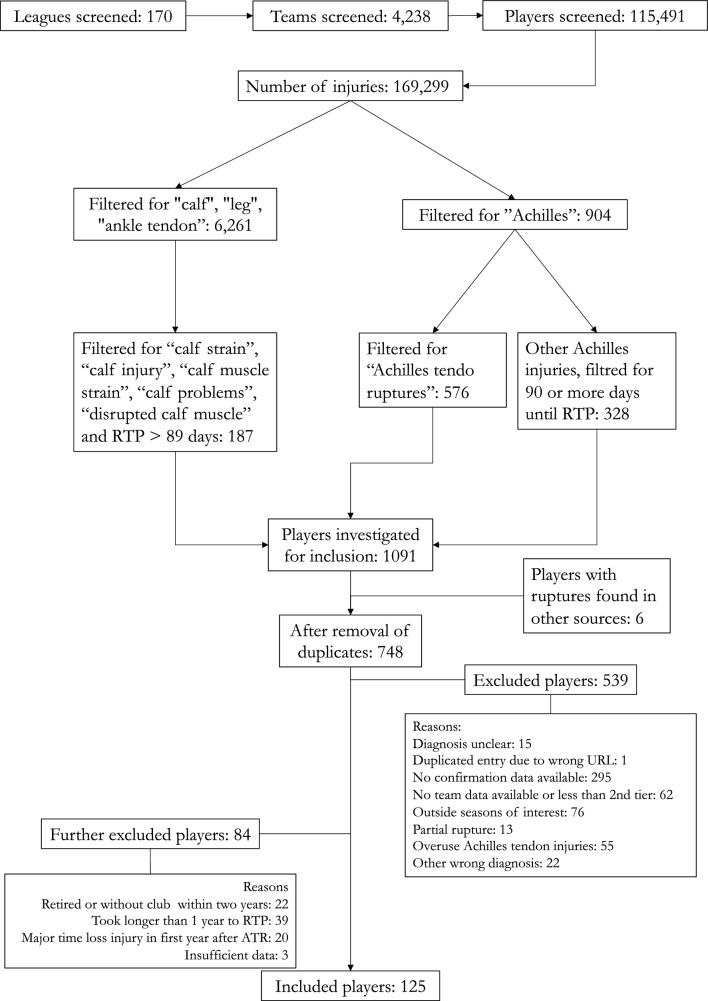
Player screening and selection flowchart, with exclusion criteria

After the exclusion criteria, 125 athletes were included in the ATR group. Detailed information about the screening and selection process, including exclusion criteria, is shown in Fig. 1. Thus, the CTRL group comprised 750 players. Player demographics and baseline characteristics can be found in Table 1.

**Table 1:** Player demographics for the Achilles tendon rupture (ATR) and matched-control (CTRL) groups

	Forwards			Midfielders			Defenders			Goalkeepers		
	ATR	CTRL	*p*	ATR	CTRL	*p*	ATR	CTRL	*p*	ATR	CTRL	*p*
*N* =	48	288		33	198		35	210		9	54	
Age, years	28.1 (5.1)	27.9 (5.1)	n.s.	28.0 (4.6)	27.7 (3.6)	n.s.	28.5 (6.0)	27.8 (4.1)	n.s.	30.5 (6.2)	29.4 (8.0)	n.s.
Height, cm	181 (10)	181 (8)	n.s.	178 (9)	180 (7)	n.s.	184 (7)	183 (10)	n.s.	183 (4)	187 (3)	n.s.

All values are expressed as medians and interquartile ranges

Statistical significance set at *p* < 0.05, *n.s.* without statistical significance

### Analysis of match participation

**Fig. 2:**
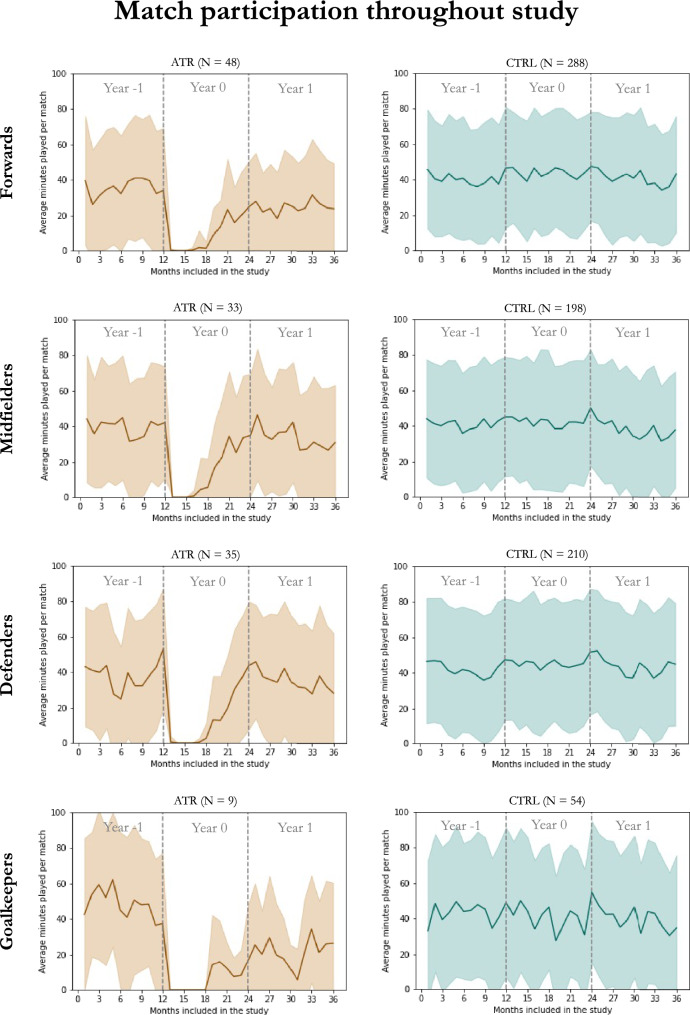
Plot of average minutes played per match (*y*-axis) for all players included throughout the study time frame and computed in 30-day intervals (*x*-axis) per group and playing position. Shaded areas correspond to standard deviation. *ATR* Achilles tendon rupture group, *CTRL* matched-control group

Data from 79,825 matches were analyzed. The number of matches available for analysis was not statistically significantly different between the ATR and CTRL groups in Year -1 through Year 1. Data related to match participation and reasons for match absence can be found in Table 2. In the ATR group, statistically significant differences between Year -1 and Year 1 were found for average MPM, matches played, matches started, and matches sat on the bench, but not for reasons regarding match absence. Statistically significant differences between Year -1 and Year 1 were found for the CTRL group regarding reasons for match absence. A plot of average MPM throughout the study time frame, computed in 30-day intervals per playing position for both groups, can be seen in Fig. 2.

**Table 2:** Comparison between the Achilles tendon rupture (ATR) and matched-control (CTRL) groups regarding match participation and reasons for match absence

	Year -1			Year 0			Year 1		
	ATR	CTRL	*p*	ATR	CTRL	*p*	ATR	CTRL	*p*
Net weighted PM metric	− 0.27 (3.11)	− 0.18 (2.44)	n.s.	− 0.16 (0.82)	− 0.30 (2.54)	n.s.	− 0.34 (1.85)	− 0.21 (2.65)	n.s.
Average MPM, minutes	46 (30)	52 (33)	n.s.	11 (18)	55 (34)	< 0.01	37 (37)	51 (34)	< 0.01
Matches sat on bench, %	6 (16)	6 (13)	n.s.	5 (9)	6(10)	n.s	9 (17)	6 (12)	0.01
Matches started, %	52 (36)	59 (38)	n.s.	12 (20)	62 (39)	< 0.01	40 (42)	60 (39)	< 0.01
Matches played, %	65 (36)	70 (32)	n.s.	21 (23)	73 (28)	< 0.01	56 (38)	71 (29)	< 0.01
Reasons for match absence									
Coach choice	100 (40)	95 (44)	n.s.	20 (23)	100 (35)	< 0.01	100 (33)	88 (61)	0.03
Medical issues	0 (33)	0 (25)	n.s.	79 (23)	0 (13)	< 0.01	0 (24)	0 (44)	n.s.
Other	0 (0)	0 (0)	n.s.	0 (0)	0 (7)	< 0.01	0 (0)	0 (0)	0.01

All values are expressed as medians and interquartile ranges

*MPM* minutes played per match

Statistical significance set at *p* < 0.05. *n.s.* without statistical significance

### Analysis of match performance

**Fig. 3:**
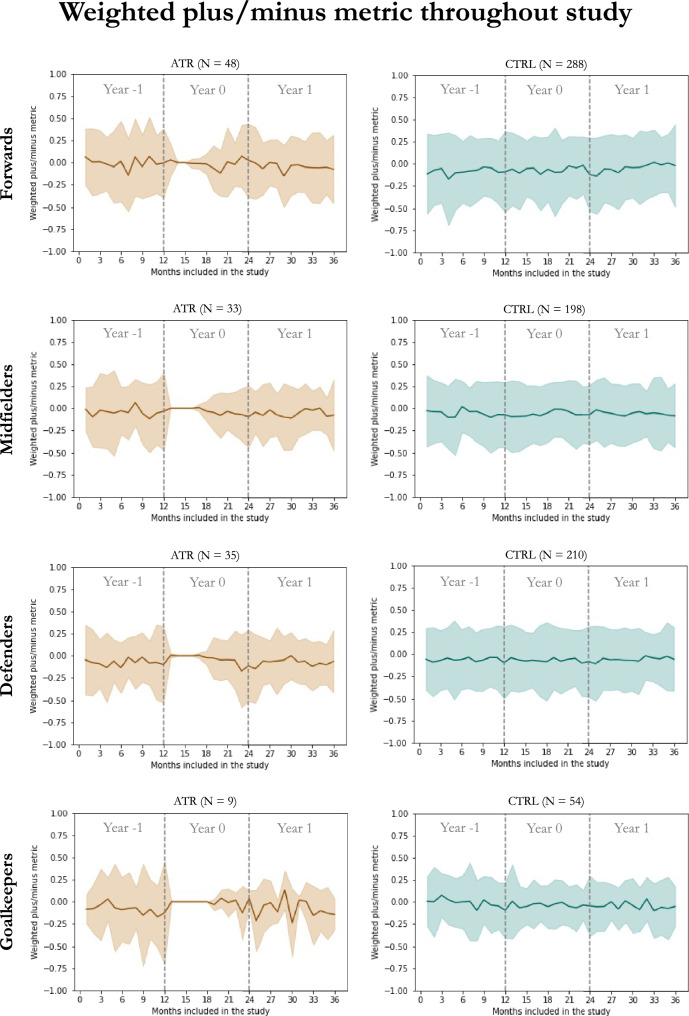
Plot of the weighted plus/minus metric [28] (*y*-axis) for all players included throughout the study time frame and computed in 30-day intervals (*x*-axis) per group and playing position. Shaded areas correspond to standard deviation. *ATR* Achilles tendon rupture group, *CTRL* matched-control group

Data from 76,464 matches were included in the performance calculation. Data were missing in 391 matches (0.5%), imputed using the player’s mean performance per match for the study period. No statistically significant differences were found regarding the net weighted PM metric in any year between the ATR and CTRL groups. Exploratory analysis of the net weighted PM metric per year, player position, and study group can be found in Table 3. No statistically significant differences were found regarding the net weighted PM metric per player position between Year -1 and Year 1. A plot of the computed metric throughout the study time frame, calculated in 30-day intervals per playing position for both groups, can be seen in Fig. 3.

**Table 3:** Comparison between the Achilles tendon rupture (ATR) and matched-control (CTRL) groups regarding match performance computed using the weighted plus/minus metric described by Schultze and Wellbrock [28]

	Year -1			Year 0			Year 1		
	ATR	CTRL	*p*	ATR	CTRL	*p*	ATR	CTRL	*p*
Forwards	0.17 (2.72)	− 0.18 (3.08)	n.s.	− 0.11 (0.94)	− 0.28 (2.42)	n.s.	− 0.10 (1.40)	− 0.05 (2.87)	n.s.
Midfielders	0.207 (3.51)	− 0.24 (2.22)	n.s.	− 0.17 (0.94)	− 0.22 (2.64)	n.s.	− 0.19 (1.59)	− 0.14 (2.47)	n.s.
Defenders	− 0.63 (2.23)	− 0.24 (2.54)	n.s.	− 0.22 (1.16)	− 0.48 (2.77)	n.s.	− 0.56 (1.66)	− 0.34 (2.69	n.s.
Goalkeepers	0.18 (2.94)	0.0 (0.99)	n.s.	− 0.14 (0.40)	− 0.30 (1.79)	n.s.	− 0.77 (1.42)	− 0.15 (1.50)	n.s.

All values are expressed as medians and interquartile ranges

Statistical significance set at *p* < 0.05. *n.s.* without statistical significance

When comparing the ATR and CTRL groups’ performance using bootstrapping to compute the Mann–Whitney *U* test statistic and along with the Hodges–Lehmann shift estimator, neither group consistently outperformed the other across the evaluated periods. In Year -1, there was no significant performance difference, with a p value of 0.8268 and a median difference of − 0.01 (95% confidence interval (CI) − 0.444 and 0.398). This trend continued in Year 0, with a p value of 0.1776 and a median difference of 0.001 (95% CI − 0.180 to 0.192). By Year 1, though a slight trend emerged with a *p* value of 0.071, the performance difference, with a median value of − 0.002 (95% CI: − 0.316 to 0.291), remained without statistical significance.

## Discussion

The objective of the present study was to assess the effect of ATRs on individual player performance using a weighted PM metric. The main finding is that no statistically significant differences were found between Year -1 and Year 1, despite a statistically significant decrease in the average MPM. Thus, the null hypothesis in this study could not be refuted.

Previous studies comparing match participation before and after ATRs have shown contradictory results, with some reporting a statistically significant reduction in MPM [2, 31], while others have not [4, 29]. Of note, a post-injury decrease in minutes played has also been observed in basketball, as has in American football [18]. Differences between studies may be attributed to how match participation was calculated and how matches and players were selected for inclusion, e.g., some studies may have only considered official matches. At the same time, others may have also included friendly matches, or some studies may have found predominantly higher profile leagues and players through searches, typically with higher pre-injury performance and, consequently, with better post-injury match participation.

Pre-injury, i.e., in Year -1, average MPMs were comparable between the ATR and CTRL groups, but not in Year 0 and Year 1, which provides confidence in the results of the present study. Although a slight downward trend was observed in the CTRL group, the negative effect of an ATR in match participation was evident [4, 29, 31]. It can be hypothesized that, for some players, the long recovery time may be a tipping point for loss of pitch time, since the player will lose their place to another teammate. Soccer is a team sport; when an athlete is injured, another athlete takes their place. The recovering player will have to outperform other players in the same position, who, by the time unrestricted practice is allowed again, have been training and competing regularly for a significant portion of the season.

Differences in the number of matches lost due to medical issues between the ATR and CTRL groups and between Year -1 and Year 1 in the ATR group were not statistically significant. Patients with previous ATRs exhibit increased knee loading during jumping and/or jogging [26, 34], increased knee range of motion and overextension of the knee on initial contact during running [14], and/or increased knee flexion and reduced hip extension [23]. On the one hand, it has been hypothesized that these changes in lower limb biomechanics, which might be influenced not only by tendon elongation following an ATR [3] but also by factors such as kinesiophobia [17] and diminished calf muscle strength, could predispose athletes to a variety of knee injuries [16, 25]. While severe knee injuries like anterior cruciate ligament (ACL) tears are possible [7], overload injuries may be more directly related to the biomechanical changes post-ATR. On the other hand, given the several potential contributing factors and the broad spectrum of knee injuries, this topic should be approached with caution. Furthermore, the present study may not have been sufficiently powered to detect subtle but significant differences in the incidence of specific knee injuries between the ATR and CTRL groups within the observed time frame. In addition, smaller differences between groups may have been undetected due to limitations regarding the reporting of injuries of lesser severity [11] and the inability to adequately adjust for exposure.

Players suffering from ATRs exhibited a return to similar performance levels as measured by a weighted PM metric. Although PM metrics have been mainly used in sports analytics, e.g., in basketball and ice hockey [15], their use in assessing return-to-performance following injury has also been reported [1, 32]. Nevertheless, PM metrics have been criticized for being too simplistic, and, thus, “adjusted” versions of these metrics have also been proposed, e.g., to account for the strengths of teammates or whether the player is in the home or away team. The PM metric used herein, chosen for its applicability to the available data and straightforwardness, adjusts a player’s contribution to team performance by accounting for the team’s winning probabilities [27]. As noted elsewhere [15], while comparing two players with a PM metric of zero, one in the top-performing team and the other in the worst team in a league, the player in the worst team probably deserves more recognition. Accordingly, one notable limitation of PM metrics is their use in comparing players from different teams [13]. However, because the main objective of the present study was to measure performance throughout time for each player, using this kind of metric was justified.

The main limitation of this study is related to the lack of ability to confirm the diagnosis, since data were gathered from a publicly available source and not from first-hand medical assessment. To account for this limitation, two researchers independently sought further evidence of the athlete sustaining an ATR, such as in club press reports and player interviews. One of the exclusion criteria was players with  an ATR who did not RTP within a year following the injury, which is another limitation of the present study. While this could introduce a selection bias, favoring those with more favorable recovery profiles, the study aimed to evaluate performance changes in players who were able to RTP within this timeframe. Future studies might consider including players with longer recovery times. Furthermore, an assessment of match participation, measured as the average minutes played per match, regardless of RTP within 1 year, has been reported in our previous work [2]. Another important limitation is the relatively small sample size for some playing positions and the lack of a sample size calculation, which may contribute to the risk of a Type II error, possibly leading to incorrect failure to refute the null hypothesis. Further research, including sample size calculations, is needed to confirm these results. Finally, limitations of the weighted PM metric used herein should be recognized, as it may not be sensitive enough to individual performance variations in specific scenarios, e.g., if the remaining team can compensate for a momentarily underperforming player. In addition, coaches may prefer to reserve players returning from prolonged recoveries to match moments where the score is *settled*, i.e., when the match is felt to be either won or lost, which will not affect the weighted PM metric.

### Implications for clinical practice and future research

The results of the present study may be used to inform athletes sustaining ATRs and help them make career decisions. Despite a deleterious effect on match participation, players suffering these injuries could return to a comparable level of performance, as measured using a weighted PM metric. Athletes and staff need to be counseled about the possibility that previously recognized long-lasting consequences of ATRs on sports participation may reflect an “opportunity cost” related to the prolonged recovery time inherent to such an injury and not necessarily loss of technical ability.

Further research may be aimed at evaluating the psychological consequences to athletes and shared beliefs of athletes and staff regarding ATRs and their implications for future physical function in sports. Research on soccer performance indicators and changes induced by injuries is also of interest. Although several key performance indicators have been proposed for soccer analytics [10, 12], their feasibility as outcome measures in medical research must be considered. Finally, epidemiological studies on the incidence of future lower limb injuries following an ATR are also needed.

## Conclusion

Despite a decrease in match participation, no differences were found between athletes suffering from ATRs and controls regarding the weighted PM metric.

## Data Availability

The data that support the findings of this study are available from the corresponding author upon reasonable request.

## References

[1] Chauhan A, Stotts J, Ayeni OR, Khan M (2021). Return to play, performance, and value of National Basketball Association players following Achilles tendon rupture. Phys Sportsmed.

[2] Diniz P, Abreu M, Lacerda D, Martins A, Pereira H, Ferreira FC, Kerkhoffs GM, Fred A (2022). Pre-injury performance is most important for predicting the level of match participation after Achilles tendon ruptures in elite soccer players: a study using a machine learning classifier. Knee Surg Sports Traumatol Arthrosc.

[3] Diniz P, Pacheco J, Guerra-Pinto F, Pereira H, Ferreira FC, Kerkhoffs G (2020). Achilles tendon elongation after acute rupture: is it a problem? A systematic review. Knee Surg Sports Traumatol Arthrosc.

[4] Forlenza EM, Lavoie-Gagne OZ, Lu Y, Diaz CC, Chahla J, Forsythe B (2021). Return to play and player performance after Achilles tendon rupture in UEFA professional soccer players: a matched-cohort analysis of players from 1999 to 2018. Orthop J Sports Med.

[5] Forsythe B, Lavoie-Gagne OZ, Forlenza EM, Diaz CC, Mascarenhas R (2021). Return-to-Play times and player performance after ACL reconstruction in elite UEFA professional soccer players: a matched-cohort analysis from 1999 to 2019. Orthop J Sports Med.

[6] Fox G, Gabbe BJ, Richardson M, Oppy A, Page R, Edwards ER, Hau R, Ekegren CL (2016). Twelve-month outcomes following surgical repair of the Achilles tendon. Injury.

[7] Grassi A, Macchiarola L, Filippini M, Lucidi GA, Della Villa F, Zaffagnini S (2020). Epidemiology of anterior cruciate ligament injury in Italian first division soccer players. Sports Health.

[8] Grassi A, Rossi G, D’Hooghe P, Aujla R, Mosca M, Samuelsson K, Zaffagnini S (2020). Eighty-two per cent of male professional football (soccer) players return to play at the previous level two seasons after Achilles tendon rupture treated with surgical repair. Br J Sports Med.

[9] Hägglund M, Waldén M, Bahr R, Ekstrand J (2005). Methods for epidemiological study of injuries to professional football players: developing the UEFA model. Br J Sports Med.

[10] Herold M, Kempe M, Bauer P, Meyer T (2021). Attacking key performance indicators in soccer: current practice and perceptions from the elite to youth academy level. J Sports Sci Med.

[11] Hoenig T, Edouard P, Krause M, Malhan D, Relógio A, Junge A, Hollander K (2022). Analysis of more than 20,000 injuries in European professional football by using a citizen science-based approach: An opportunity for epidemiological research?. J Sci Med Sport.

[12] Hughes M, Caudrelier T, James N, Donnelly I, Kirkbride A, Duschesne C (2012). Moneyball and soccer—an analysis of the key performance indicators of elite male soccer players by position. J Hum Sport Exerc.

[13] Hvattum LM (2019). A comprehensive review of plus-minus ratings for evaluating individual players in team sports. Int J Comput Sci Sport.

[14] Jandacka D, Silvernail JF, Uchytil J, Zahradnik D, Farana R, Hamill J (2017). Do athletes alter their running mechanics after an Achilles tendon rupture?. J Foot Ankle Res.

[15] Kharrat T, McHale IG, Peña JL (2020). Plus–minus player ratings for soccer. Eur J Oper Res.

[16] Krosshaug T, Nakamae A, Boden BP, Engebretsen L, Smith G, Slauterbeck JR, Hewett TE, Bahr R (2007). Mechanisms of anterior cruciate ligament injury in basketball: video analysis of 39 cases. Am J Sports Med.

[17] Kvist J, Silbernagel KG (2022). Fear of movement and reinjury in sports medicine: relevance for rehabilitation and return to sport. Phys Ther.

[18] LaPrade CM, Chona DV, Cinque ME, Freehill MT, McAdams TR, Abrams GD, Sherman SL, Safran MR (2022). Return-to-play and performance after operative treatment of Achilles tendon rupture in elite male athletes: a scoping review. Br J Sports Med.

[19] Lavoie-Gagne O, Mehta N, Patel S, Cohn MR, Forlenza E, Nwachukwu BU, Forsythe B (2021). Adductor muscle injuries in UEFA soccer athletes: a matched-cohort analysis of injury rate, return to play, and player performance from 2000 to 2015. Orthop J Sports Med.

[20] Lemme NJ, Li NY, DeFroda SF, Kleiner J, Owens BD (2018). Epidemiology of Achilles tendon ruptures in the United States: athletic and nonathletic injuries from 2012 to 2016. Orthop J Sports Med.

[21] Leventer L, Eek F, Hofstetter S, Lames M (2016). Injury patterns among elite football players: a media-based analysis over 6 seasons with emphasis on playing position. Int J Sports Med.

[22] McHale IG, Scarf PA, Folker DE (2012). On the development of a soccer player performance rating system for the english premier league. Interfaces.

[23] Mezzarobba S, Bortolato S, Giacomazzi A, Fancellu G, Marcovich R, Valentini R (2012). Percutaneous repair of Achilles tendon ruptures with Tenolig: quantitative analysis of postural control and gait pattern. Foot.

[24] Parekh SG, Wray WH, Brimmo O, Sennett BJ, Wapner KL (2009). Epidemiology and outcomes of Achilles tendon ruptures in the National Football League. Foot Ankle Spec.

[25] Podraza JT, White SC (2010). Effect of knee flexion angle on ground reaction forces, knee moments and muscle co-contraction during an impact-like deceleration landing: implications for the non-contact mechanism of ACL injury. Knee.

[26] Powell HC, Silbernagel KG, Brorsson A, Tranberg R, Willy RW (2018). Individuals post achilles tendon rupture exhibit asymmetrical knee and ankle kinetics and loading rates during a drop countermovement jump. J Orthop Sports Phys Ther.

[27] Schultze SR, Wellbrock C-M (2018). A weighted plus/minus metric for individual soccer player performance. J Sports Anal.

[28] Slagers AJ, van Veen E, Zwerver J, Geertzen JHB, Reininga IHF, van den Akker-Scheek I (2021). Psychological factors during rehabilitation of patients with Achilles or patellar tendinopathy: a cross-sectional study. Phys Ther Sport.

[29] Sochacki KR, Jack RA, Hirase T, McCulloch PC, Lintner DM, Varner KE, Cosculluela PE, Harris JD (2019). There is a high return to sport rate but with reduced career lengths after Achilles tendon repair in Major League Soccer players. J ISAKOS.

[30] Transfermarkt.com. Football transfers, rumours, market values, news and statistics

[31] Trofa DP, Noback PC, Caldwell J-ME, Miller JC, Greisberg JK, Ahmad CS, Vosseller JT (2018). Professional soccer players’ return to play and performance after operative repair of Achilles tendon rupture. Orthop J Sports Med.

[32] Van Pelt KL, Lapointe AP, Galdys MC, Dougherty LA, Buckley TA, Broglio SP (2019). Evaluating performance of national hockey league players after a concussion versus lower body injury. J Athl Train.

[33] Walker J, Nicholson G, Jongerius N, Parelkar P, Harris N, Bissas A (2020). Commonly reported isokinetic parameters do not reveal long-term strength deficits of the Triceps surae complex following operative treatment of Achilles tendon rupture. J Biomech.

[34] Willy RW, Brorsson A, Powell HC, Willson JD, Tranberg R, Grävare Silbernagel K (2017). Elevated knee joint kinetics and reduced ankle kinetics are present during jogging and hopping after Achilles tendon ruptures. Am J Sports Med.

